# Supplementary material for: A Critical View of the Application of the APEX Software (Aqueous Photochemistry of Environmentally-Occurring Xenobiotics) to Predict Photoreaction Kinetics in Surface Freshwaters
Source: Molecules. 2019 Dec 18;25(1):9. doi: 10.3390/molecules25010009 (PMC7017383; doi:10.3390/molecules25010009)
Supplement: Supplementary file 1 [file molecules-25-00009-s001.zip › 3.2.4_gcc-4.4.0.zip!/3.2.4_gcc-4.4.0/3.2.4_gcc-4.4.0/doc/octave/html/interpreter/Entering-Debug-Mode.html]

Entering Debug Mode - Untitled


Next: Leaving Debug Mode,
Up: Debugging


---

### 13.1 Entering Debug Mode

There are two basic means of interrupting the execution of an Octave
script. These are breakpoints see Breakpoints, discussed in the next
section and interruption based on some condition.

Octave supports three means to stop execution based on the values set in
the functions `debug_on_interrupt`, `debug_on_warning` and
`debug_on_error`.

— Built-in Function: val = **debug\_on\_interrupt** ()  
— Built-in Function: old\_val = **debug\_on\_interrupt** (new\_val)  
> Query or set the internal variable that controls whether Octave will try
> to enter debugging mode when it receives an interrupt signal (typically
> generated with `C-c`). If a second interrupt signal is received
> before reaching the debugging mode, a normal interrupt will occur.

— Built-in Function: val = **debug\_on\_warning** ()  
— Built-in Function: old\_val = **debug\_on\_warning** (new\_val)  
> Query or set the internal variable that controls whether Octave will try
> to enter the debugger when a warning is encountered.

— Built-in Function: val = **debug\_on\_error** ()  
— Built-in Function: old\_val = **debug\_on\_error** (new\_val)  
> Query or set the internal variable that controls whether Octave will try
> to enter the debugger when an error is encountered. This will also
> inhibit printing of the normal traceback message (you will only see
> the top-level error message).
